# Polyporus Polysaccharide Ameliorates Bleomycin-Induced Pulmonary Fibrosis by Suppressing Myofibroblast Differentiation *via* TGF-β/Smad2/3 Pathway

**DOI:** 10.3389/fphar.2020.00767

**Published:** 2020-05-26

**Authors:** Jintao Jiang, Fang Wang, Aishu Luo, Shiyu Lin, Xiaoke Feng, Wei Yan, Yumeng Shi, Qian Zhang, Xin Gu, Guoliang Cui, Jianan Wang, Lei Wang, Qiande Zhang, Wenfeng Tan

**Affiliations:** ^1^ Institute of Integrated Chinese and Western Medicine, Nanjing Medical University, Nanjing, China; ^2^ Division of Rheumatology, the First Affiliated Hospital of Nanjing Medical University, Nanjing, China; ^3^ Division of Cardiology, the First Affiliated Hospital of Nanjing Medical University, Nanjing, China; ^4^ Division of Rheumatology, Yancheng First People's Hospital, Yancheng, China

**Keywords:** polyporus polysaccharide, pulmonary fibrosis, systemic sclerosis, myofibroblast, TGF-β/Smad2/3

## Abstract

Pulmonary fibrosis is a major cause of morbidity and mortality in systemic sclerosis (SSc) with no effective medication. Polyporus polysaccharide (PPS), extracted from Chinese herbs, has immune regulation, anticancer, antioxidant and antiinflammatory activities. This study aims to investigate antifibrotic effects of PPS. We show that PPS markedly ameliorates bleomycin-induced lung fibrosis in mice. Myofibroblasts are the effector cells responsible for excessive deposition of extracellular matrix (ECM) proteins in fibrotic diseases. *In vitro* evidence reveals that PPS exerts potent antifibrotic effects by inhibiting fibroblast-to-myofibroblast transition, suppressing ECM deposition, and repressing lung fibroblast proliferation and migration. We also find that PPS inhibits TGF-β1-induced Smad2/3 activating. This study is the first to demonstrate an antifibrotic role of PPS in lungs, thus warranting further therapeutic evaluation.

## Introduction

Pulmonary Fibrosis is a common pathological feature of systemic sclerosis (SSc). Clinically significant pulmonary fibrosis develops in over 20% SSc patients while high-resolution computed tomography (HRCT) detects interstitial changes in 90% patients (Solomon et al., 2013). Pulmonary fibrosis is associated with progressive decline in pulmonary function and reduced survival, accounting for 33% deaths of patients (Wells, 2014). High extent lung fibrosis is also one of the major causes of pulmonary hypertension, other severe and life threatening complications of SSc (Saygin and Domsic, 2019). Current immunosuppressive therapy for SSc exerts its effects mainly on inflammatory process and therefore has limited benefits on the fibrotic process, rendering the search for a novel antifibrotic strategy an urgent need (Distler et al., 2019b).

The molecular basis of pulmonary fibrosis in SSc remains unclear. However, a number of fibrosis related pathways or cellular effectors have been described as promising therapy targets (Distler et al., 2019b). Fibrosis is characterized by excessive deposition and remodeling of the extracellular matrix (ECM) (Knudsen et al., 2017). Transforming growth factor (TGF)-β is current considered as a central mediator in activation of the fibrotic program (Györfi et al., 2018). Upon TGF-β stimulation, fibroblasts are activated and undergo phenotypic transition into myofibroblasts, a key effector cell in fibrotic process (van Caam et al., 2018). Alpha-smooth muscle actin (α-SMA) is a useful marker for myofibroblast differentiation and activation (Gu et al., 2007). Persistent myofibroblast activation could secrete excessive collagen, and fibronectin in the lung interstitium, resulting in progressive fibrosis and loss of lung function (van Caam et al., 2018). Therefore, targeting TGF-β signaling or myofibroblast differentiation has been considered as a useful strategy for developing new antifibrotics.

To date, Pirfenidone (PFD) and Nintedanib are the only approved antifibrotic drugs for the treatment of SSc pulmonary fibrosis and idiopathic pulmonary fibrosis (Khanna et al., 2016; Flaherty et al., 2018; Distler et al., 2019a). Pirfenidone could suppress TGF-β expression thereby attenuating fibroblast proliferation, myofibroblast differentiation, and collagen and fibronectin synthesis (Oku et al., 2008). As a tyrosine kinase inhibitor, Nintedanib targets the receptors platelet-derived growth factor receptor α and β, fibroblast growth factor receptor (FGFR) 1–3, and vascular endothelial growth factor receptor (VEGFR) that inhibits fibroblast proliferation (Sato et al., 2017). Although Pirfenidone and Nintedanib could ameliorate SSc-pulmonary fibrosis in some clinical studies (Khanna et al., 2016; Flaherty et al., 2018; Distler et al., 2019a), the adverse side effects, expensive costs, and relatively weak effect on preventing or reversing fibrosis progression emphasize a great unsatisfied need in treatment.

ZhuLing (Polyporus) has been a widely used medicinal fungus in China for more than 2,500 years. The pharmaceutical components in Polyporus umbellatus include polysaccharides, ergosterols, biotin, proteins, and other molecules (Guo et al., 2019). Among them, Polyporus polysaccharide (PPS) is the major bioactive of polyporus umbellatus. The main component in PPS is a β-glucan with a (1-3)-β-glucose back-bone and (1-6)-β-glucose side chains and a molecular weight of approximately 1.6 × 10^5^ Da (Zong et al., 2012). Previous study has reported a variety of pharmacological activities of PPS including immune regulation, anticancer, antioxidant, and antiinflammatory and hepatoprotective activities (Li et al., 2010). Interestingly, many kinds of polysaccharides have shown antifibrotic ability to improve pulmonary, liver and renal fibrosis (Chen et al., 2018; Li et al., 2019; Shan et al., 2019). We therefore hypothesize that PPS might have the same effect for treating SSc related pulmonary fibrosis. In present study, we evaluated the potential antifibrotic effect of PPS in both bleomycin (BLM)-induced pulmonary fibrosis model and activated lung fibroblasts, and revealed the underlying mechanism.

## Materials and Methods

### Cell Line and Reagents

The human lung fibroblasts (HLFs) cell line and fibroblast medium were purchased from ScienCell (Catalog #3310, California, USA). HLFs are isolated from nonfibrotic or fibrotic (or scleroderma) adult human lung tissue. Pirfenidone (PFD) was purchased from Meilun Bio-Technology (Dalian, China). Bleomycin Hydrochloride (BLM) was purchased from Nippon Kayaku (Massachusetts, United States). Recombinant human transforming growth factor β1 (TGF-β1) was purchased from Peprotech (California, USA). Masson trichrome staining kits were purchased from Solarbio Science & Technology (Beijing, China). Cell Counting Kit-8 was purchased from Dojindo (Kyushu, Japan). PrimerScript™RT reagent kit was purchased from TaKaRa (Dalian, China). Power SYBR Green PCR Master Mix was purchased from Applied Biosystems (Carlsbad, CA, USA). Primary antibodies and secondary antibodies are listed in **Tables S1** and **S2**.

### Preparation of PPS

**Figure 1:**
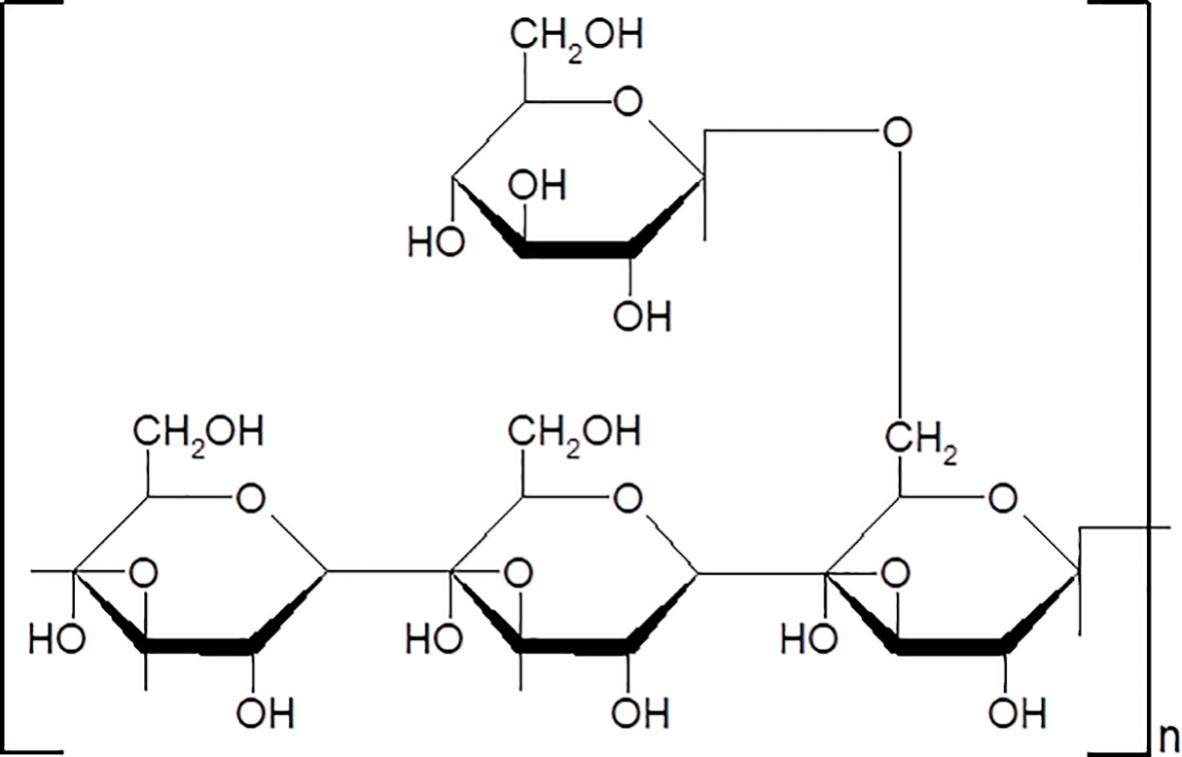
The chemical structure of Polyporus polysaccharide (PPS).

PPS were purchased from Yuanye Bio-Technology (Shanghai, China) (Lot. Number: C08A9Y57961). The purity of PPS is 98%. The chemical structure of PPS was shown in **Figure 1**. PPS is a water-soluble polysaccharide. In present study, PPS was dissolved in distilled water or cell culture medium and filtered through a 0.22μM membrane for experimental use.

### BLM-Induced Pulmonary Fibrosis Model

Male C57BL/6 mice (6–8 weeks old, weighting 18–22 g) were purchased from Qinglongshan Animal Cultivation Farm (Nanjing, China). All experiments were performed in accordance with the China Animal Welfare Legislation and were approved by Welfare Ethics Review Committee of Nanjing Medical University. Mice were randomly and averagely divided into four subgroups: Control group, BLM group (model group), BLM+PFD group (positive group), and BLM+PPS group. For inducing pulmonary fibrosis model, mice were treated with a single dose of chemical grade BLM (50 μl at 5 U/kg, Thermo Fisher Scientific, USA) *via* intratracheal infusion. Control group mice received the same volume of normal saline instead of BLM. According to our preliminary experiment results, a dosage of 100 mg/kg PPS was used in animal experiment (**Figure S1**). After modeling, the BLM+PPS group mice were administrated with PPS (100 mg/kg) by intraperitoneal injection (IP injection) and BLM+PFD group mice were intraperitoneally administrated with PFD (50 mg/kg). The BLM group mice were given IP injection of the same amount of normal saline. All the above drugs were administered once a day for 21 days. At day 21, all mice were sacrificed and the lung tissues were collected for further examination described below.

### Histopathological Evaluation

For histopathology examination, left lung tissue was fixed in 4% paraformaldehyde solution, dehydrated with ethanol, embedded in paraffin. Sections of 4 μm were taken and stained with by hematoxylin and eosin (HE) and Masson's trichrome (Masson) to investigate levels of lung inflammation and collagen deposition. Three slides in each mouse and 10 fields in each slide were examined for grading inflammatory and fibrotic lesions. The severity of the pulmonary fibrosis was assessed based on Ashcroft's scoring system (0 = normal; 1 = minimal fibrotic thickening of alveolar walls; 2 = moderate thickening of walls without obvious damage to lung architecture; 3 = increased fibrosis with definite damage to lung structure and formation of fibrous bands or small fibrous masses; 4 = severe distortion of structure and large fibrous areas) (Ashcroft et al., 1988). The two independent observers who scored all histology samples were blinded to the treatment.

### Immunohistochemistry

Lung tissue sections were deparaffinized using xylene, rehydrated in a graded ethanol series. After antigen retrieval, sections were incubated with 3% hydrogen peroxide for 10min to quench the endogenous peroxidase, and blocked with 5% BSA. Lung sections were incubated with rabbit anti-α-SMA antibody (1:500 dilution) at 4°C overnight. Then, the slices were incubated with HRP-Goat-Anti-Rabbit IgG secondary antibody for 1 h at 37°C. The color reaction was then made with HRP-linked polymer detection system and counterstained with hematoxylin.

### Western Blot

Proteins extracted from either lung tissues or cells were analyzed by Western blot as described in previous studies (Wang et al., 2019). Briefly, HLFs or lung tissues were lysed in RIPA lysis buffer containing with protease, phosphatase inhibitor and phenylmethylsulfonyl fluoride (PMSF). Cell lysates/tissue homogenates were resolved on 10% SDS-PAGE gels and transferred to polyvinylidene fluoride (PVDF) membrane. The membranes were blocked with 5% nonfat dry milk powder/BSA in TBS containing 0.1% Tween-20. Antibodies used in this study included those specific for fibronectin, collagen type 1, collagen type 3, α-SMA, Smad2/3, TGF-β receptor I and II, and phospho-Smad2/3. Primary antibodies and secondary antibodies using in Western blot analysis are listed in **Table S1**. GAPDH antibody was used as an internal reference. The percentage of target protein band density to GAPDH density was calculated and densitometrically analyzed by Image J software (NIH, Bethesda, MD).

### Cell Culture, Cell Proliferation, and Morphological Analysis

The HLFs were cultured in fibroblast medium (FM, ScienCell Research Laboratories, Carlsbad, CA) consisted of a proprietary basal medium formulation supplemented with 2% fetal bovine serum (FBS), 1% fibroblast growth supplement, and 1% penicillin/streptomycin and placed in an incubator at 37°C with 5% CO^2^ atmosphere. Cells were serum-starved for 24 h in fibroblast medium with 0% FBS before treatment. For cell proliferation assay, HLFs were incubated in fresh serum-free medium and Cell Counting kit-8 (CCK-8) was used to determine cell proliferation as described in previous studies (Huang et al., 2017; Shi et al., 2019). Cell morphology was observed and randomly captured under Nikon inverted microscope.

### Immunofluorescence Staining

After different treatment for 48 h, adherent cells were fixed in 4% paraformaldehyde for 20 min, and then permeabilized in 0.2% Triton X-100 and blocked with 3% BSA in PBS for 60 min at room temperature. The fixed cells were incubated with Rabbit anti-α-SMA antibody overnight at 4°C. Goat anti-rabbit IgG secondary antibody was added to samples for 2 h incubation at room temperature. Then DAPI was used to stain nuclei. Immunofluorescence was observed and images were captured using Zeiss microscope.

### Cell Migration Analysis

HLFs were seeded at confluent status and the cell monolayer was scratched with a 200-µl pipette tip. After treated with culture medium alone (Con), 1 mg/ml of PPS or 0.5 mg/ml PFD for 24 and 48 h, the migratory cells in the gap were counted using a light microscope at ×100 magnification. All of the data were obtained in three independent experiments.

### RNA Extraction and Real-Time Quantitative PCR

Total RNA was extracted using TRIzol reagent (Invitrogen, Carlsbad, CA, USA) and cDNA was synthesized by the RNA PCR Core Kit (Applied Biosystems, Branchburg, NJ, USA). Levels of gene expression were quantified by SYBR Green real-time PCR using an ABI Prism 7900 Sequence Detection System. The sequences of the primers were listed in **Table S3**. Relative expression was calculated with normalization to GAPDH values by using the ^2ΔΔ^ Ct method. The primer sequences were summarized in **Table S3**.

### Statistical Analysis

The data analysis was performed by using GraphPad Prism 8 Software. Data are shown as the average ( ± SD) of at least three independent experiments. Significance of differences among the groups was assessed by one-way ANOVA and Tukey's test. Significant difference of means was declared at *p* < 0.05.

## Results

### PPS Alleviates BLM-Induced Pulmonary Fibrosis

**Figure 2:**
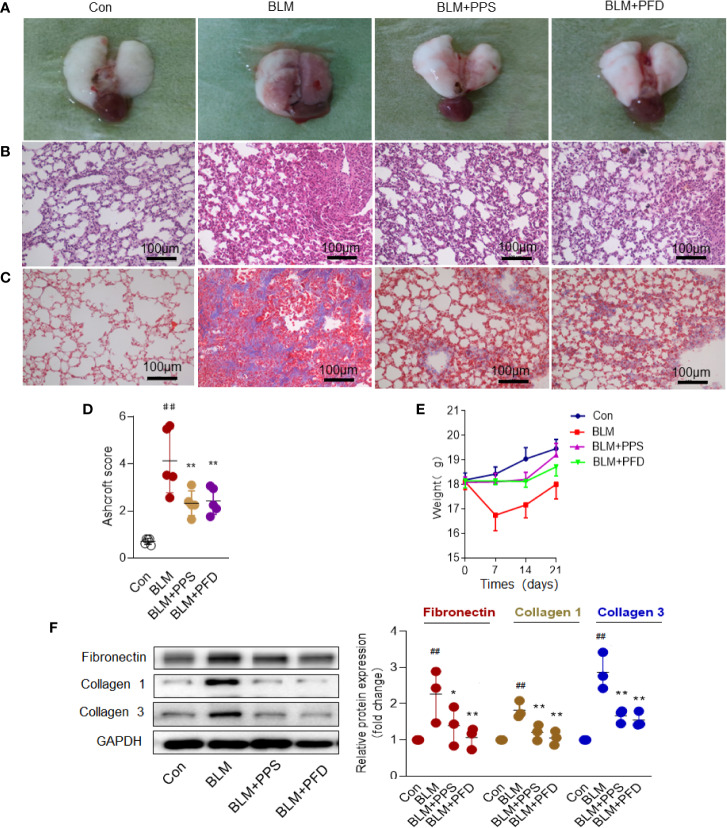
Polyporus polysaccharide (PPS) alleviates bleomycin (BLM)-induced pulmonary fibrosis. **(A)** Representative photographs of whole lungs (left lung) obtained on day 21 after treatment. **(B)** Lung fibrosis in mice, assessed by hematoxylin and eosin (HE) staining and Masson's trichrome blue staining **(C)** (Scale bars, 100 μm). Photomicrographs are representative of at least five independent experiments. **(D)** Semiquantitative analysis of pulmonary fibrosis in mice, using the Ashcroft score. Scatter plots show the mean± SD (n = 5, per group). **(E)** Wight changes in different group (n = 5, per group). **(F)** Representative Western blot results for Fibronectin, Collagen 1, and Collagen 3 expression in lung tissue. Scatter plots show the mean ± SD quantitative results obtained using three independent studies. ^##^ = *p* < 0.01 versus control (Con); * = *p* < 0.05, ** = *p* < 0.01 versus BLM group.

The model of BLM-induced lung fibrosis is the most used experimental model in rodents (Tashiro et al., 2017). To test whether PPS could prevent BLM-induced pulmonary fibrosis, mice were exposed to BLM or saline by intratracheal infusion, and subsequently administrated with PPS (100 mg/kg), PFD (50 mg/kg) or vehicle daily starting the day of BLM administration. Morphologically, BLM treated animals showed collapsed and haemorrhagic lungs with rough surfaces and gray fibrous nodules gray at day 21 (**Figure 2A**). PPS treatment group and PFD group (positive group) did not exhibit appreciable changes in morphology, as compared with control mice (**Figure 2A**). Histologically, in the BLM group, obvious thickened alveolar walls, destruction of alveolar space and infiltration of inflammatory cells within interstitial tissue were observed. Similar with PFD group, PPS administration led to a markedly improved lung structure and decreased inflammatory cells infiltration (**Figures 2B, C**).

The overall level of fibrotic changes was assessed quantitatively based on the Ashcroft scoring system (Bogatkevich et al., 2011). The score in BLM treated mice was nearly 4-fold higher than that in control mice. PPS and PFD significantly reduced the fibrotic score by 48.9% and 49.1%, respectively, compared with the BLM plus placebo group (**Figure 2D**). BLM group had a markedly weight loss compared to the control group, but BLM-induced weight loss was reversed by PPS or PFD treatment (**Figure 2E**).

### PPS Improves Deposition of ECM and α-SMA Expression in BLM-Induced Pulmonary Fibrosis

Excessive deposition of ECM plays a key role in pulmonary fibrosis process (Knudsen et al., 2017). Collagen is the most prevalent protein in the ECM. Masson staining was first used to detect the deposition of collagen in the lung tissue. In the BLM group, the distorted lung structure presented with dramatically increased blue collagen staining, while in the PPS or PFD treated group, reduced collagen deposition with a partial recovery of lung morphology were found (**Figure 2C**). By Western blot, we further confirmed that PPS or PFD treatment remarkably reduced BLM-induced ECM proteins expression, including Fibronectin, Collagen 1, and Collagen 3 (**Figure 2F**).

**Figure 3:**
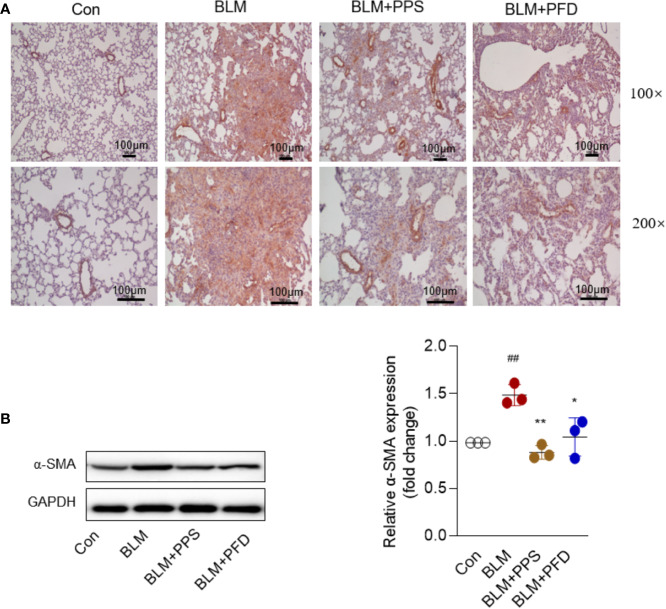
Polyporus polysaccharide (PPS) decreases alpha-smooth muscle actin (α-SMA) expression in bleomycin (BLM)-induced pulmonary fibrosis. **(A)** Lung tissue sections from BLM-treated mice stained for α-SMA. Photomicrographs are representative of five mice (black scale bars represent 100 µm at ×100 and ×200 magnification, respectively). **(B)** Representative Western blot results for α-SMA expression in lung tissue, normalized against GAPDH. The graph shows the results of the densitometric quantification of the Western blot analysis, which are presented as fold changes as compared with control group. Scatter plots show the mean ± SD quantitative results obtained using 3 independent studies. ^##^ = p < 0.01 versus control (Con); * = p < 0.05, ** = p < 0.01 versus BLM group.

α-SMA is a recognized marker of activated fibrogenic cells of myofibroblasts and is also often used as a marker to evaluate the therapeutic effect of antifibrotic drugs. At day 21, immunohistochemistry indicated that positive staining with anti-α-SMA antibodies was markedly increased in lung tissue from mice treated with bleomycin (**Figure 3A**), whereas it was significantly reduced in PPS and PFD group. We also measured α-SMA expression by Western blot, the consistent results were found that α-SMA protein expression was dramatically inhibited by PPS or PFD treatment, compared to BLM group treatment with saline (p < 0.01) (**Figure 3B**).

Together, these results indicate that PPS improves BLM-induced pulmonary fibrosis.

### PPS Inhibits Myofibroblast Differentiation

Myofibroblasts are considered as an important effector cell of tissue fibrogenesis. Following activation by TGF-β or other profibrotic stimuli, fibroblasts that differentiate to myofibroblasts have been considered as a critical step in the pathogenesis of pulmonary fibrosis. We then intend to investigate whether PPS influenced myofibroblast differentiation.

**Figure 4:**
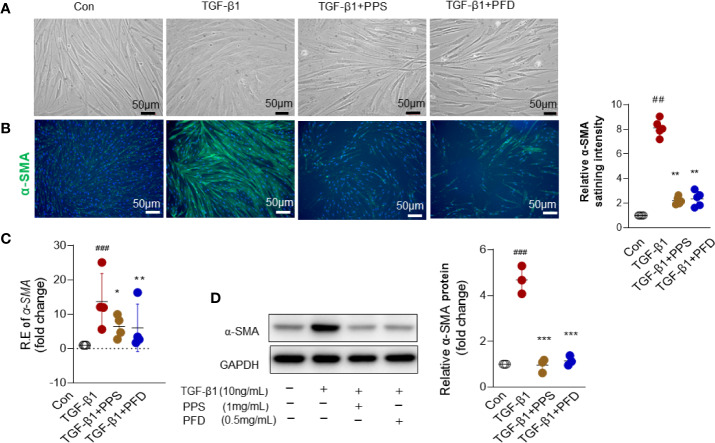
Polyporus polysaccharide (PPS) reduces alpha-smooth muscle actin (α-SMA) expression in transforming growth factor β1 (TGF-β1) treated human lung fibroblasts (HLFs). **(A)** Microscopy images are representative morphology analysis in TGF-β1 treated HLFs from four independent experiments (Scale bars, 50 μm). **(B)** Representative results for detection of alpha-smooth muscle actin (α-SMA) fluorescence in TGF-β1 treated HLFs (Scale bars, 50 μm). DAPI was used as a nuclear counterstain. Quantification of fluorescence intensity normalized to DAPI (right). **(C)** α-SMA mRNA expression in TGF-β1 treated HLFs (n = 4, per group). **(D)** Representative Western blot results for α-SMA expression TGF-β1 treated HLFs, normalized against GAPDH. The graph shows the results of the densitometric quantification of the Western blot analysis, which are presented as fold changes as compared with control group. Scatter plots show the mean ± SD quantitative results obtained from three independent studies on 3 different days. ^##^ = p < 0.01, ^###^ = p < 0.001 versus Con; * = p < 0.05, ** = p < .001, *** = p < 0.001 versus TGF-β1 treated alone.

HLFs were treated with TGF-β1 (10 ng/ml) for 48 h. We did not observe a morphology shift in fibroblasts after TGF-β treatment (**Figure 4A**). α-SMA is a typical marker of myofibroblast differentiation. We then analyzed the effect of PPS on expression of α-SMA *in vitro*. Fluorescence staining demonstrated decreased α-SMA staining in the fibroblasts cotreated with PPS or PFD and TGF-β, as compared with TGF-β1 treatment alone (**Figure 4B**). qRT-PCR (**Figure 4C**) and western-blot (**Figure 4D**) analysis indicated that α-SMA expression was significantly increased in HLFs after TGF-β1 stimulation compared with control group, whereas treatment with PPS or PFD markedly downregulated TGF-β1–induced α-SMA expression.

**Figure 5:**
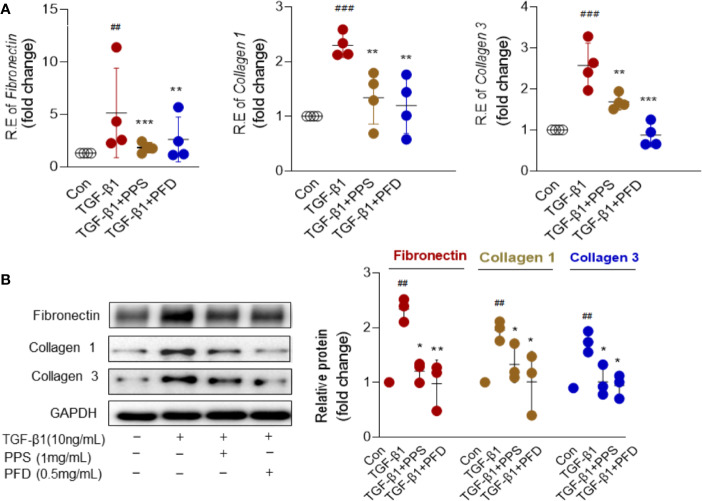
Polyporus polysaccharide (PPS) inhibits extracellular matrix (ECM) proteins expression in transforming growth factor β1 (TGF-β1) treated human lung fibroblasts (HLFs). **(A)** Levels of Fibronectin, Collagen 1, and Collagen 3 mRNA expression in TGF-β1 treated HLFs (n= 4, per group). Relative mRNA expression was calculated using ^2ΔΔ^ Ct method. The graphs are presented as fold changes as compared with control group. **(B)** Representative Western blot results for Fibronectin, Collagen 1, and Collagen 3 expression in TGF-β1 treated HLFs (n= 3, per group), normalized against GAPDH. The graph shows the results of the densitometric quantification of the Western blot analysis, which are presented as fold changes as compared with control group. Scatter plots show the mean ± SD quantitative results obtained from three independent studies on 3 different days. ^##^ = p < 0.01, ^###^ = p < 0.001 versus Con; * = p < 0.05, ** = p < 0.01, *** = p < 0.001 versus TGF-β1 treated alone.

The activated myofibroblasts have high synthetic capacity for ECM proteins. As expected, ECM components included Fibronectin, Collagen 1 and Collagen 3 were significantly increased in HLFs at mRNA (**Figure 5A**) and protein levels (**Figure 5B**) after TGF-β1 treatment compared with control group. Importantly, these components were significantly attenuated by PPS or PFD treatment (**Figures 5A, B**). We tested the effect of PPS on myofibroblast differentiation on primary HLFs that obtained from adjacent normal lung of a patient with nonsmall cell lung cancer using Thy1+ (eBioscience, San Diego, CA) flow sorting. Similar results were shown in **Figure S2**. PPS could markedly inhibit α-SMA (**Figure S2A**) and ECM components (**Figure S2B**) included Fibronectin, Collagen 1, and Collagen 3 expression in TGF-β1 treated primary HLFs. We also tested the MMP-2 and MMP-9 proteins in HLFs after TGF-β1 stimulation. As expected, TGF-β1 induced high expression of MMP-2 and MMP-9 could markedly inhibit by PPS or PFD (**Figure S3**). Our data indicated that PPS could inhibit the differentiation of the myofibroblast.

Toll-like receptors (TLRs) has emerged as a key driver of persistent fibrotic response in SSc (Frasca and Lande, 2020). We tested TLR4 expression in lung tissue from BLM treated mice by Western blot and in TGF-β1 treated HLFs by real-time PCR. TLR4 protein expression increased in the lung tissue of BLM treated mice; but TLR4 levels did not markedly change in PPS treated mice (**Figure S4A**). TLR4 mRNA expression showed an increasing trend in HLFs cell line after TGF-β1 stimulation but did not reach statistic difference in our data. Accordingly, different concentrations of PPS treatment (0.1, 0.5, 1, and 5 mg/ml) had no effect on TLR4 mRNA expression in HLFs (**Figure S4B**).

### PPS Inhibits TGF‐β1-Induced Proliferation and Migration of HLFs

**Figure 6:**
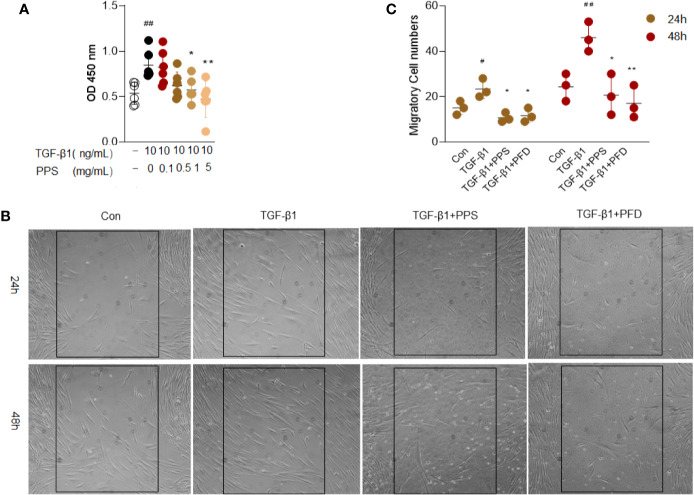
Polyporus polysaccharide (PPS) inhibits transforming growth factor β1 (TGF-β1)–induced human lung fibroblasts (HLFs) proliferation and migration. **(A)** Cell viability after different treatments was detected using a CCK-8 Kit. **(B)** Representative image is the scratch assay was used to measure fibroblast migration after TGF-β1 treated 24 and 48 h, respectively (Scale bars, 50 μm). **(C)** Scatter plots show the mean ± SD quantitative results obtained from three independent scratch assays on 3 different days. ^#^ = p < 0.05, ^##^ = p < 0.01 versus Con; * = p < 0.05, ** = p < 0.01 versus TGF-β1 treated alone.

The proliferation and migration of fibroblasts are postulated to be one of the major contributors to pulmonary fibrosis. We then investigated the effects of PPS on TGF‐β1-induced proliferation and migration of HLFs. According to the CCK-8 assay results, the absorbance of HLFs cells significantly increased after 10 ng/ml TGF-β1 stimulation. Concentrations of 1 and 5 mg/ml PPS treatment could markedly reduce the absorbance, and the optimum concentration of PPS was 1 mg/ml (**Figure 6A**). We carried out cellular toxicity by measuring the release of lactate dehydrogenase (LDH) in HLFs after PPS treatment. Results indicated that PPS showed no significant cytotoxicity on cultured HLFs (data not shown). These results demonstrate that PPS significantly inhibits HLFs cell proliferation.

Next, the scratch assay was used to measure the effect of PPS on fibroblast migration. Administration of PPS or PFD significantly inhibited TGF-β1-induced migration of HLFs, as compared with control (**Figures 6B, C**). These results showed that PPS inhibited TGF-β1-induced fibroblast proliferation and migration.

### PPS Attenuated Myofibroblast Differentiation by Suppression TGF-β/Smad Signaling

**Figure 7:**
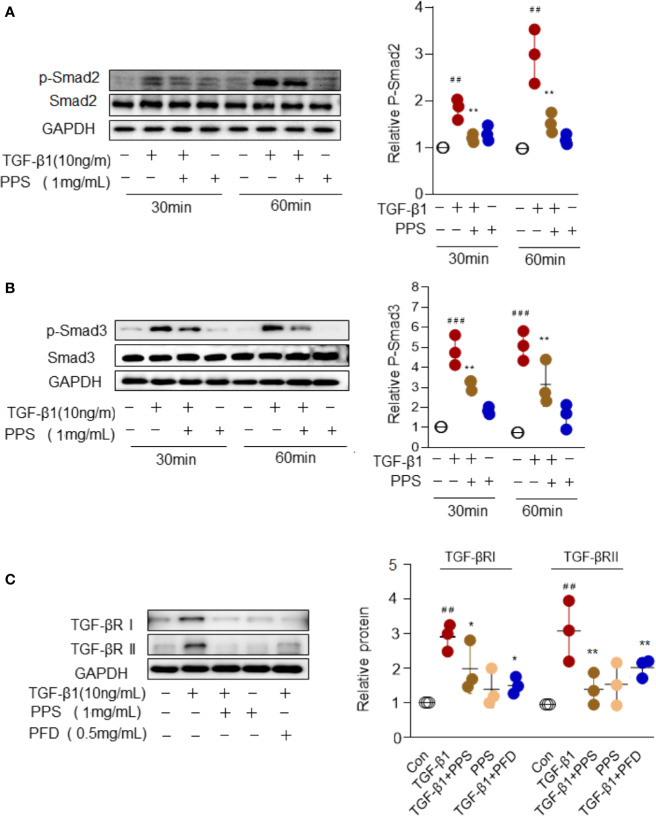
Polyporus polysaccharide (PPS) attenuates myofibroblast differentiation *via* Smad2/3 signaling. **(A, B)** Representative Western blot results for Smad2 **(A)** and Smad3 **(B)** phosphorylation in transforming growth factor β1 (TGF-β1)–treated human lung fibroblasts (HLFs). Scatter plots show the mean ± SD quantitative results obtained from three independent studies. **(C)** Representative Western blots (left) and semiquantification (n=3, right) for the blots showing levels of TGF-βRI and TGF-βRII expression in TGF‐β1-treated HLFs. ^##^ = p < 0.01, ^###^ = p < 0.001 versus Con; * = p < 0.05, ** = p < 0.01 versus TGF-β1 treated alone. Western blots were normalized against GAPDH. The graph shows the results of the densitometric quantification of the Western blot analysis, which are presented as fold changes as compared with control group.

Smad signal transduction pathways are crucial in mediating TGF-β response in fibroblasts (Györfi et al., 2018). To gain insights into the mechanisms underlying PPS inhibition of the differentiation of myofibroblast, we examined the activities of the Smad signaling pathway in HLFs upon TGF-β1 stimulation. Indeed, TGF-β stimulation for 30 to 60 min significantly induced Smad2/3 signal activation, while PPS treatment significantly attenuated p-Smad2/3 phosphorylation (**Figures 7A, B**). TGF-β exerts its function *via* its receptors I and II (TGF-βRI and TGF-βRII) (Györfi et al., 2018). Our results also revealed a direct role of PPS and PFD on inhibiting TGF-βRI and TGF-βRII expression in HLFs after TGF-β1 treatment (**Figure 7C**). Collectively, our data suggested that administration of PPS attenuated myofibroblast differentiation by repressing TGF-β/Smad2/3 signaling.

## Discussion

Pulmonary fibrosis is a major cause of morbidity and mortality in SSc. To date, only two drugs (Pirfenidone and Nintedanib) were approved for treatment pulmonary fibrosis, but showed a partial role on reducing disease progression and cannot cure the disease. This study is the first to demonstrate that PPS improves pulmonary fibrosis in mouse models. *In vitro* studies show the mechanism might be ascribed to suppress myofibroblast differentiation *via* TGF-β/Smad3 signaling pathways. Our data suggests that PPS could serve as a promising candidate for SSc-related pulmonary fibrosis therapy in clinical settings.

The biological activity of PPS has primarily focused on its antitumor and immunoregulation properties (Zhang et al., 2011; Huang et al., 2019). Our data revealed the antipulmonary fibrosis effect of PPS. BLM induced pulmonary fibrosis model was used in current study, which resembles aspects of human pulmonary fibrosis (Gilhodes et al., 2017). Twenty-one days after BLM administration, significantly thickened alveolar walls, collapsed alveolar space and collagen deposition in the lung were observed. In contrast, pulmonary fibrosis was significantly attenuated by PPS treatment. Moreover, antifibrotic effects of PPS were further evidenced by Ashcroft score, ECM protein expression and deafferentation of myofibroblasts in our data.

Previous studies indicated that PPS could ameliorate collagen deposition in renal tissue and improve renal fibrosis in a unilateral urethral obstruction induced renal fibrosis mice model (Li et al., 2019), supporting our findings of antifibrotic activity in PPS. Importantly, PPS displayed almost similar therapeutic effect with Pirfenidone on attenuating pulmonary fibrosis in our animal model and *in vitro* studies, highlighting the efficacy and feasibility of PPS as an attractive novel antifibrotic agent for clinical trials in SSc patients with interstitial lung disease.

The classical paradigm of pulmonary fibrosis is derived from a pathological consequence of persistent activation of TGF-β signaling (Györfi et al., 2018), which subsequently activation multiple key fibrogenic pathways. Among them, differentiation of resident fibroblasts into myofibroblast is a critical event of pulmonary fibrosis. Notably, our evidence showed that PPS could directly inhibit fibroblast-myofibroblast transition. Myofibroblasts are characterized by increased expression of α-SMA and collagen secretion. In present study, the addition of PPS markedly reverted increased α-SMA and collagen production in HLFs after TGF-β1 stimulation. These observations strengthen the notion that PPS has an antifibrotic effect.

In the present study TGF-β1 showed a potent ability to drive fibroblast differentiation, highlighting its key role in activation of the fibrotic program. TGF-β elicit signals through its transmembrane receptor TβRI and TβRII, and intracellular signals transducers Smad2 and Smad3. In response to TGF-β ligands, TβRII transphosphorylates TβRI, in turn mediates phosphorylation of Smad2 and Smad3. Once phosphorylated, Smad2/3 associated with Smad4 translocates to the nucleus to regulate of target gene transcription (Györfi et al., 2018; Ahmadi et al., 2019). We therefore assessed the effects of PPS on TGF-β signaling activation. First, PPS treatment markedly inhibited TβRI and TβRII expression in TGF-β1 treated HLFs. As expected, we observed that TGF-β1 triggers Smad2/3 phosphorylation, which leads to HLFs activation. PPS treatment significantly attenuated p-Smad2/3 phosphorylation. We proposed that PPS ameliorated fibroblast-myofibroblast transition at least by suppression TGF-β/Smad2/3 signaling. Further studies are needed to reveal the exact mechanism of PPS on TGF‐β signaling whether through directly interacting with TβRI/II to decrease the binding between TβRI/II and Smad2/3 or acts independently on Smad2/3.

PPS possess a variety of biological activities including the antiinflammatory effect (He et al., 2017). Inflammation also contributes to fibrosis process. In our BLM-induced pulmonary fibrosis model, infiltration of inflammatory cells within interstitial tissue was observed. PPS administration led to a markedly decreased inflammatory cells infiltration. Although our invitro data show the markedly antifibrotic effects of PPS on lung fibroblasts, indeed, we can't exclude PPS meditated-antifibrotic effects were attributed to its antiinflammatory effect. We also reveals its effect on inhibiting MMP-2 and MMP-9 expression, which are responsible for cleaving ECM (Bauvois, 2012). In addition, dysregulation of the innate immune system plays an important role in SSc. Recently, TLRs has emerged as a key driver of persistent fibrotic response in pathologies of SSc (Frasca and Lande, 2020). Numerous studies have demonstrated the ability of PPS on activation macrophages and dendritic cell through TLR4 signaling pathways (Zhang et al., 2015; Liu et al., 2017). Although we failed to detect the effect of PPS on TLR4 expression in lung tissue from BLM treated mice and in TGF-β stimulated HLFs cell line, we can't exclude western-blot and PCR are limited in their ability to catch TLR changes on certain cell types or tissues due to the complexity TLR signaling. Thus, it is possible that in addition to acting on myofibroblast and TGF-β1 signaling, PPS might exert its antifibrotic effects through modulating innate immune system *via* TLRs signaling pathways. Take gatherer, PPS might have a multifaceted mechanism of action in pulmonary fibrosis. Further studies will be needed to clarify the precise mechanism.

In conclusion, present study provides clear evidence that PPS has potential antifibrotic effects in pulmonary fibrosis animal model and HLFs. Our results offer an experimental basis for further clinical trials with PPS in SSc pulmonary fibrosis.

## Data Availability Statement

The raw data supporting the conclusions of this article will be made available by the authors, without undue reservation, to any qualified researcher.

## Ethics Statement

The animal study was reviewed and approved by Welfare Ethics Review Committee of Nanjing Medical University.

## Author Contributions

WT, JJ, FW, and QiandeZ were involved in the design of the study. AL, SL, XG, XF, QianZ, JW, LW, YS, GC, and WY were involved in the conduct of the study. JJ and XF undertook analysis, and all authors were involved in interpretation of the data. WT, JJ, and FW prepared the manuscript and all authors were involved in the review and approval of the final manuscript.

## Conflict of Interest

The authors declare that the research was conducted in the absence of any commercial or financial relationships that could be construed as a potential conflict of interest.

## Abbreviations

α-SMA, α-Smooth muscle actin; BLM, bleomycin; ECM, extracellular matrix; FGFR, fibroblast growth factor receptor; HE, hematoxylin and eosin; HLFs, human lung fibroblasts; HRCT, high-resolution computed tomography; PFD; Pirfenidone; PPS, Polyporus polysaccharide; SSc, systemic sclerosis; TGF-β, transforming growth factor-β; TGF-β1, recombinant human transforming growth factor β1; TGF-βRI and TGF-βRII, TGF-β receptor I and II.
