# Supplementary figures and images for: Polyporus Polysaccharide Ameliorates Bleomycin-Induced Pulmonary Fibrosis by Suppressing Myofibroblast Differentiation via TGF-β/Smad2/3 Pathway
Source: Front Pharmacol. 2020 May 26;11:767. doi: 10.3389/fphar.2020.00767 (PMC7264095; doi:10.3389/fphar.2020.00767)

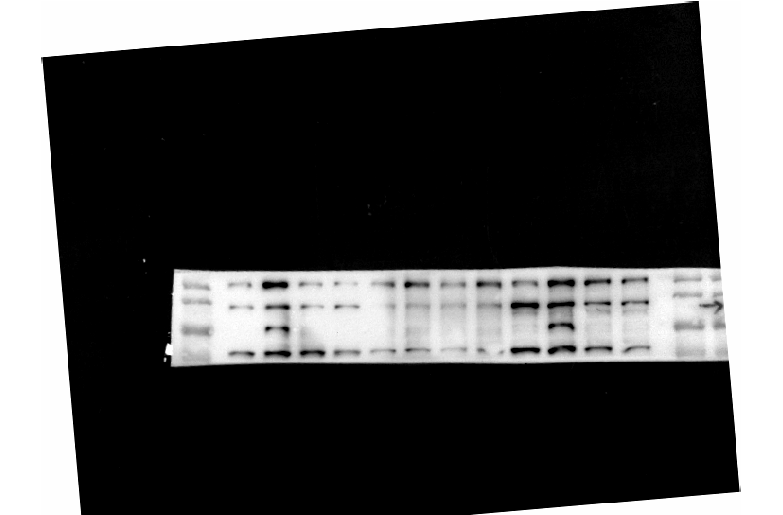

Supplement: Supplementary file 1 [file DataSheet_1.zip › Original images of blots修改/FIG2 F/Collagen 1.tif]

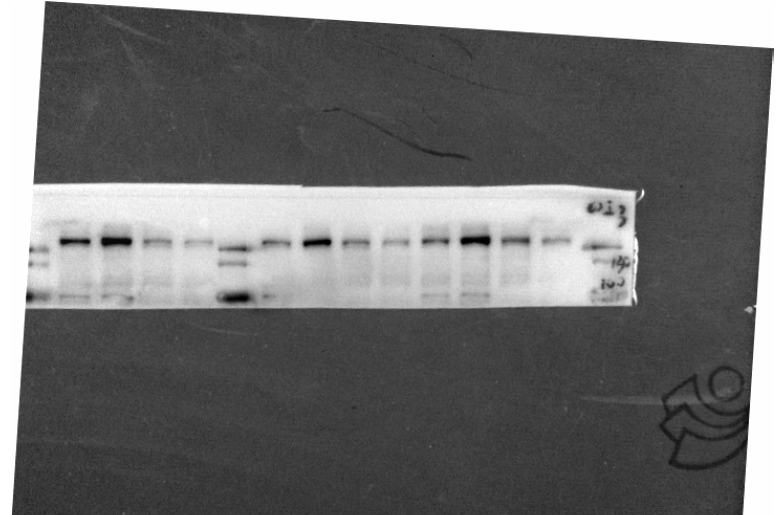

Supplement: Supplementary file 1 [file DataSheet_1.zip › Original images of blots修改/FIG2 F/Collagen 3.tif]

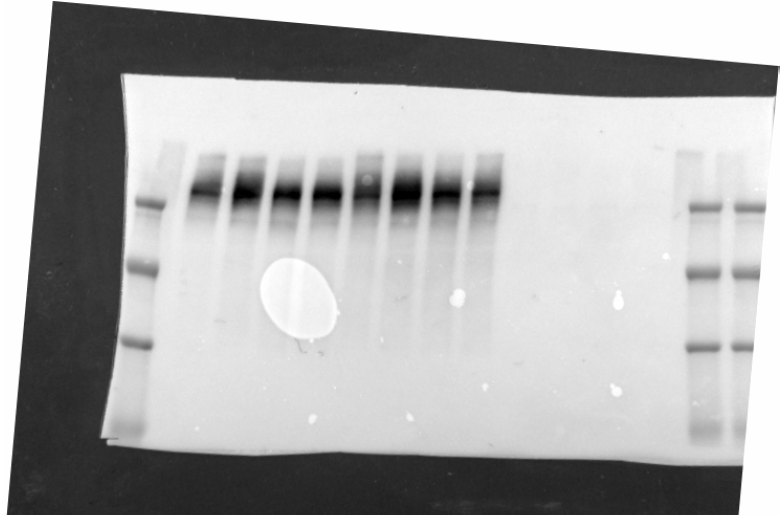

Supplement: Supplementary file 1 [file DataSheet_1.zip › Original images of blots修改/FIG2 F/Fibronectin.tif]

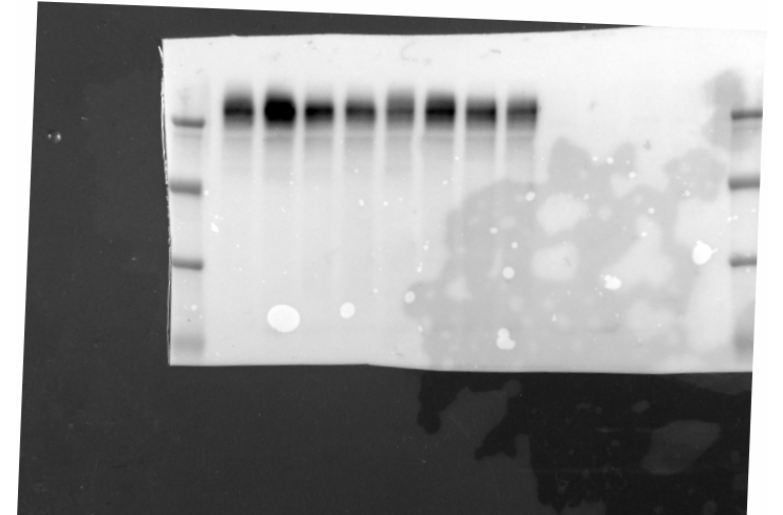

Supplement: Supplementary file 1 [file DataSheet_1.zip › Original images of blots修改/FIG2 F/Fibronectin2.tif]

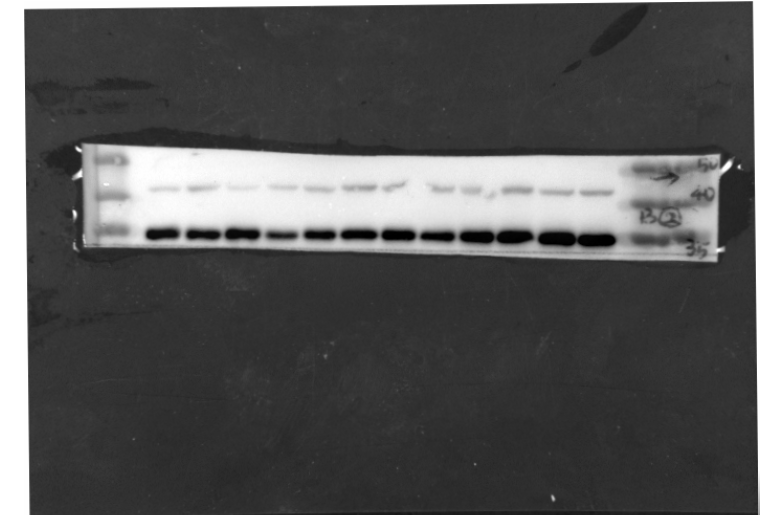

Supplement: Supplementary file 1 [file DataSheet_1.zip › Original images of blots修改/FIG2 F/GAPDH.tif]

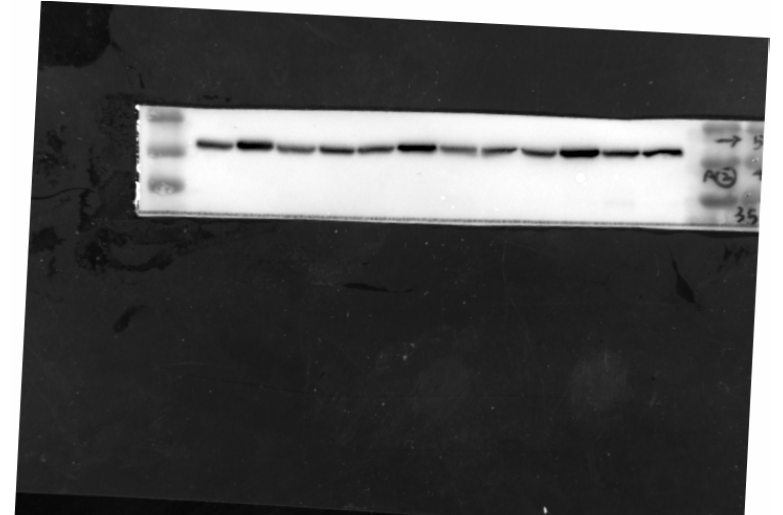

Supplement: Supplementary file 1 [file DataSheet_1.zip › Original images of blots修改/FIG3 B/α-SMA.tif]

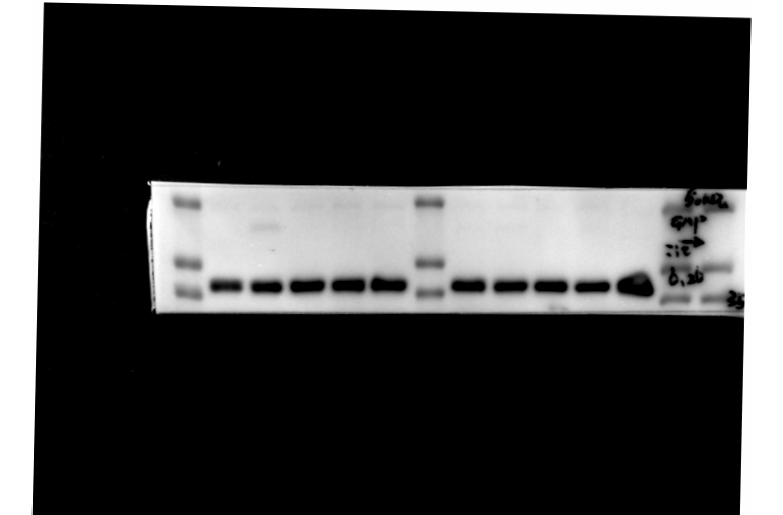

Supplement: Supplementary file 1 [file DataSheet_1.zip › Original images of blots修改/FIG4 D/GAPDH2.tif]

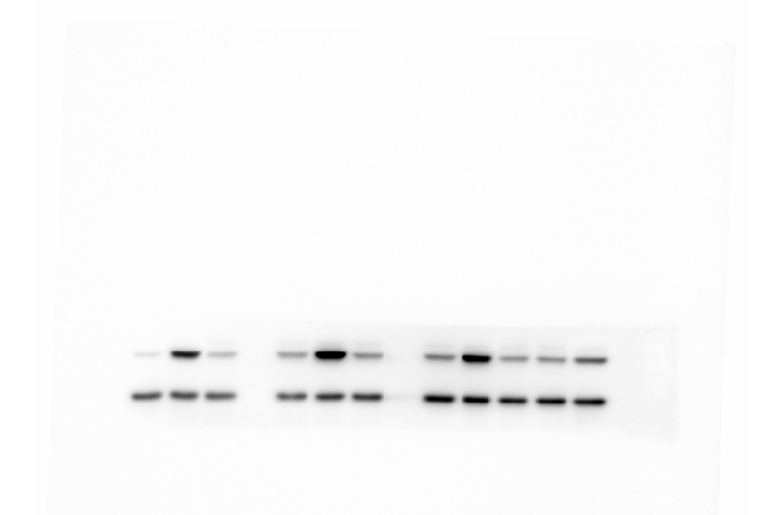

Supplement: Supplementary file 1 [file DataSheet_1.zip › Original images of blots修改/FIG4 D/SMA.tif]

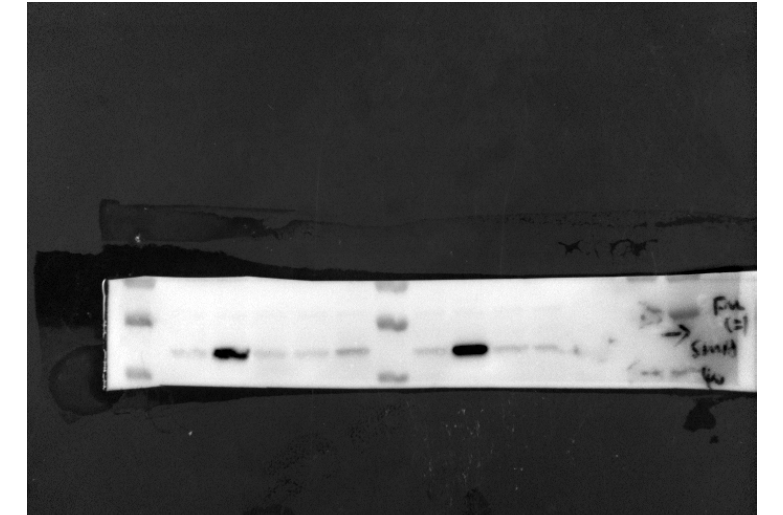

Supplement: Supplementary file 1 [file DataSheet_1.zip › Original images of blots修改/FIG4 D/α-SMA-2.tif]

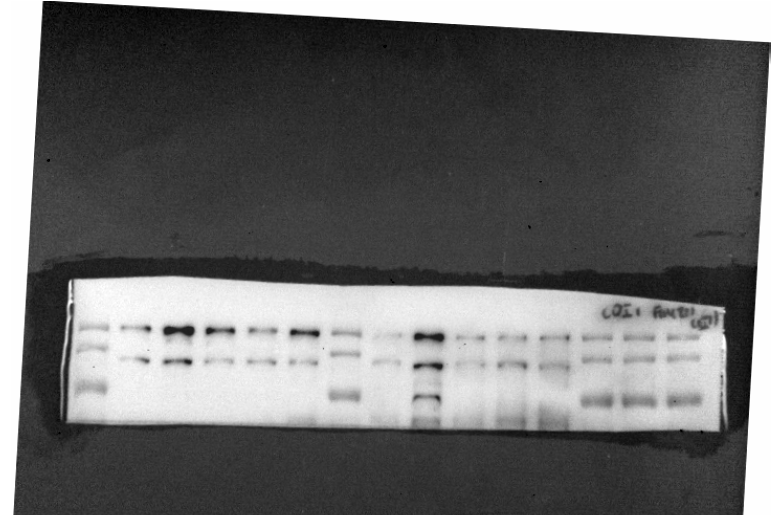

Supplement: Supplementary file 1 [file DataSheet_1.zip › Original images of blots修改/FIG5 B/Collagen 1.tif]

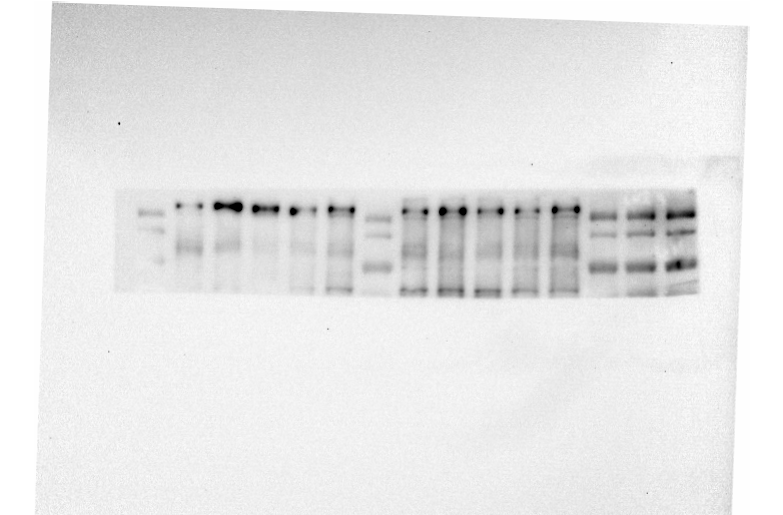

Supplement: Supplementary file 1 [file DataSheet_1.zip › Original images of blots修改/FIG5 B/Collagen 3.tif]

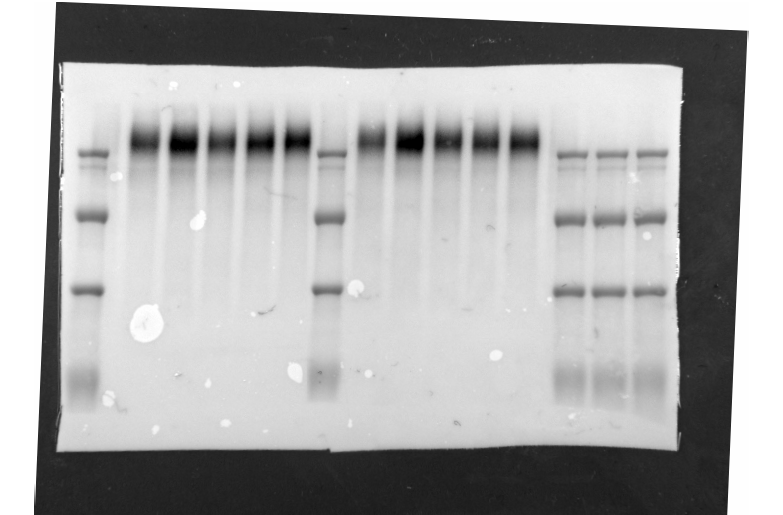

Supplement: Supplementary file 1 [file DataSheet_1.zip › Original images of blots修改/FIG5 B/Fibronectin.tif]

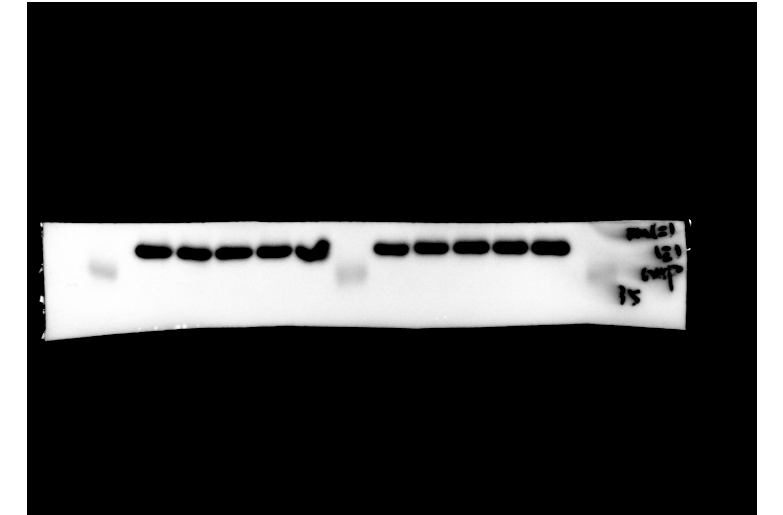

Supplement: Supplementary file 1 [file DataSheet_1.zip › Original images of blots修改/FIG5 B/GAPDH4.tif]

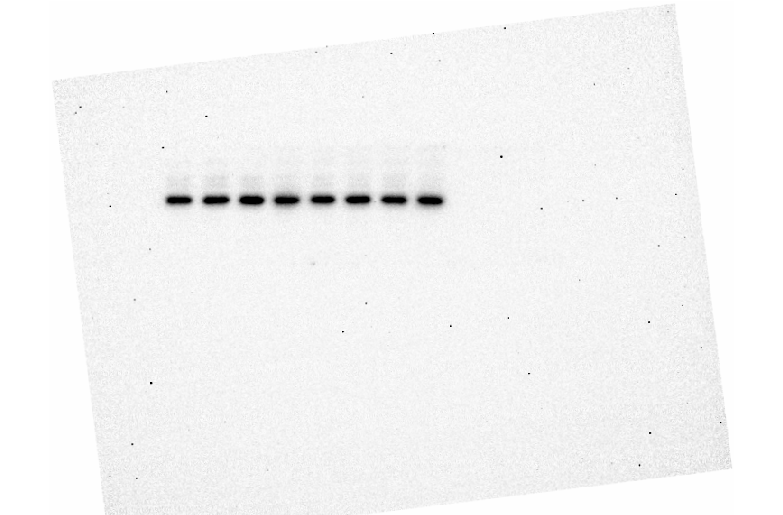

Supplement: Supplementary file 1 [file DataSheet_1.zip › Original images of blots修改/FIG7 A/GAPDH.tif]

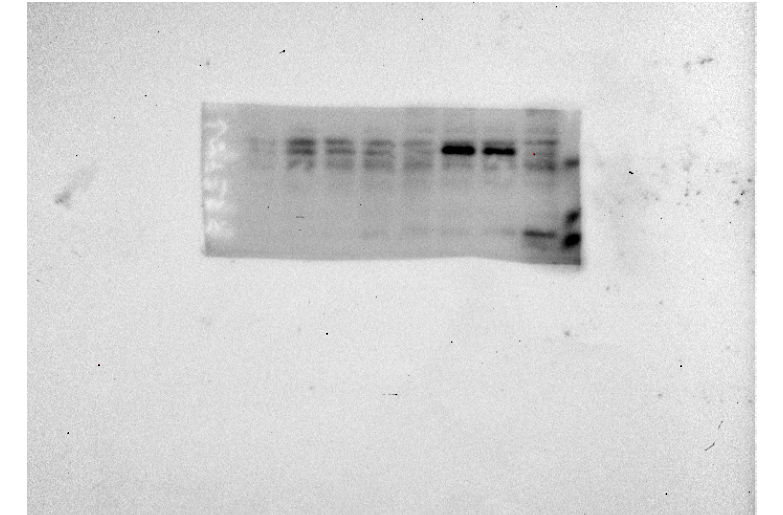

Supplement: Supplementary file 1 [file DataSheet_1.zip › Original images of blots修改/FIG7 A/p-Smad2.tif]

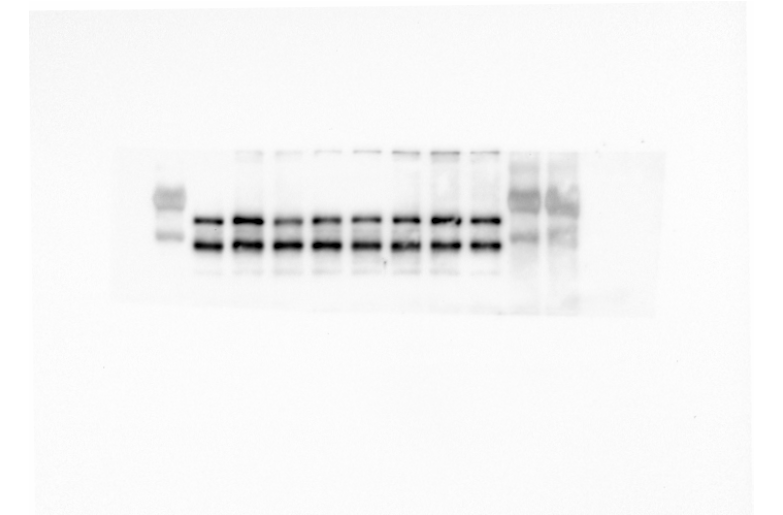

Supplement: Supplementary file 1 [file DataSheet_1.zip › Original images of blots修改/FIG7 A/Smad2.tif]

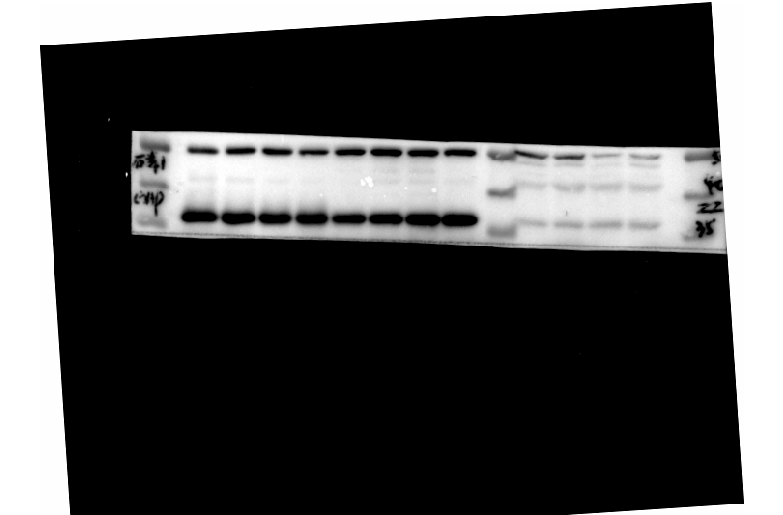

Supplement: Supplementary file 1 [file DataSheet_1.zip › Original images of blots修改/FIG7 B/GAPDH.tif]

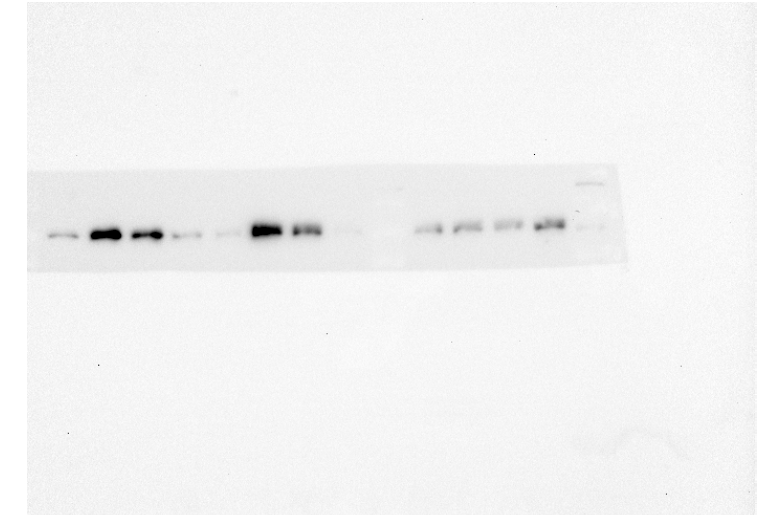

Supplement: Supplementary file 1 [file DataSheet_1.zip › Original images of blots修改/FIG7 B/P-Smad3.tif]

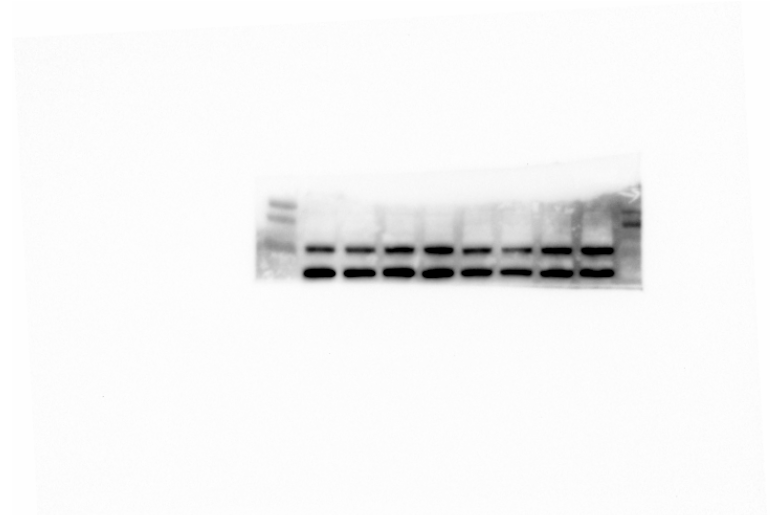

Supplement: Supplementary file 1 [file DataSheet_1.zip › Original images of blots修改/FIG7 B/SMAD3.tif]

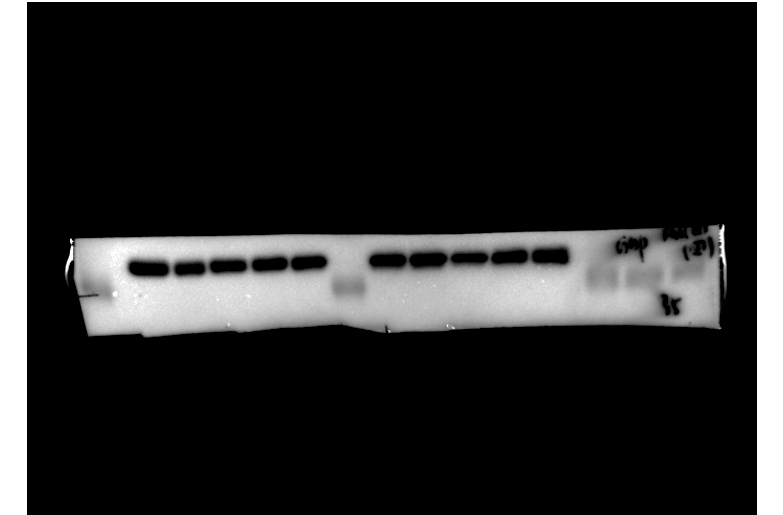

Supplement: Supplementary file 1 [file DataSheet_1.zip › Original images of blots修改/FIG7 C/TGFRI/GAPDH3.tif]

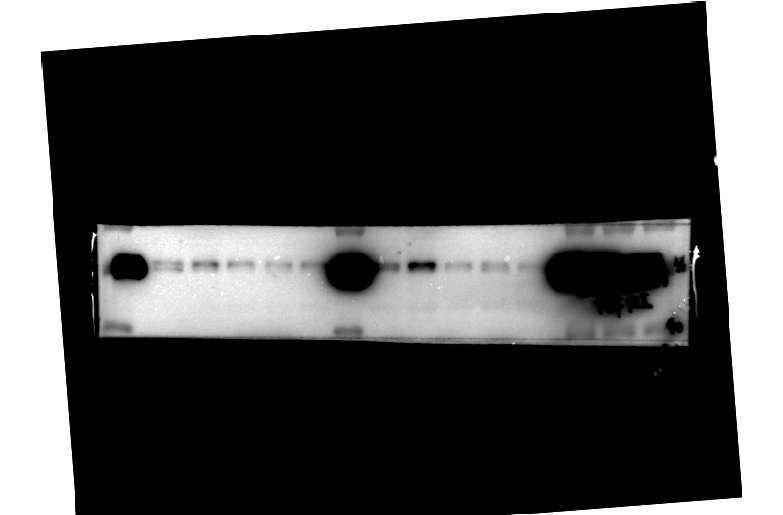

Supplement: Supplementary file 1 [file DataSheet_1.zip › Original images of blots修改/FIG7 C/TGFRI/TGF-RI.tif]

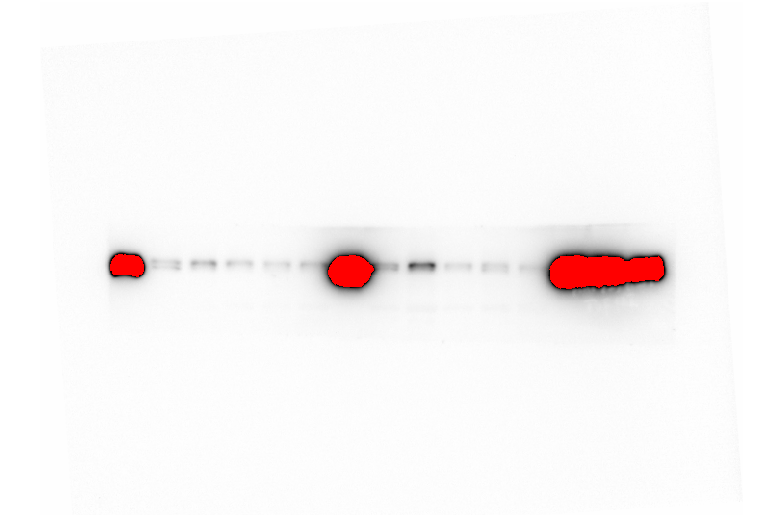

Supplement: Supplementary file 1 [file DataSheet_1.zip › Original images of blots修改/FIG7 C/TGFRI/TGFR1-1.tif]

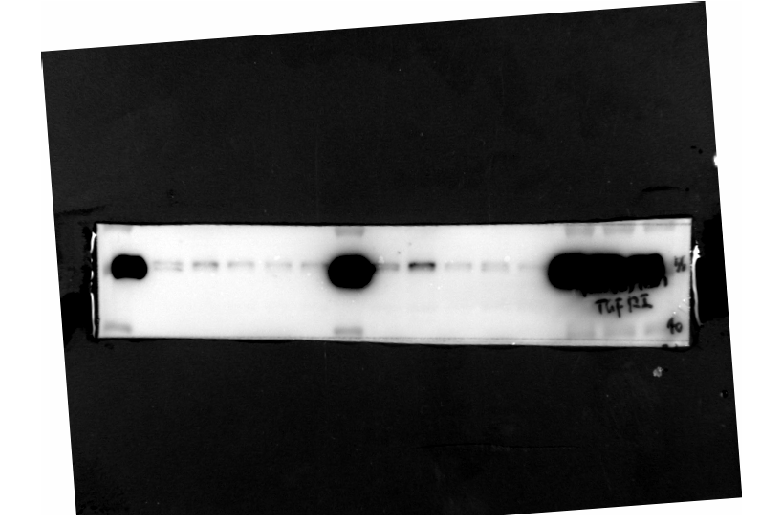

Supplement: Supplementary file 1 [file DataSheet_1.zip › Original images of blots修改/FIG7 C/TGFRI/TGFRI.tif]

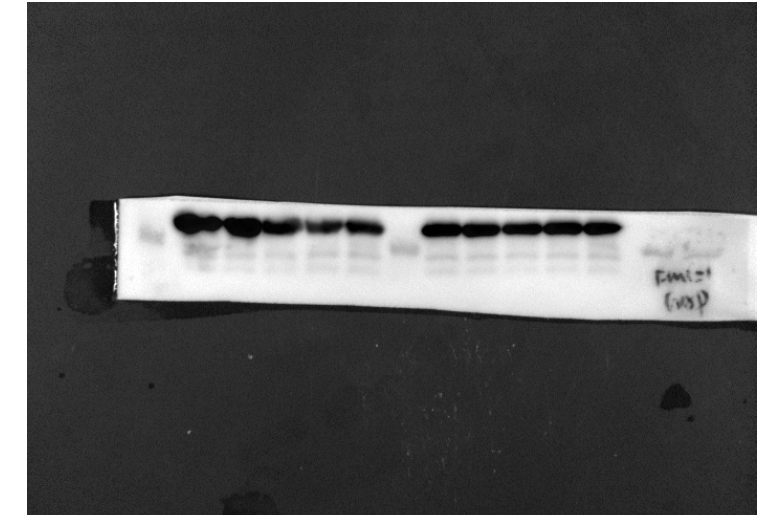

Supplement: Supplementary file 1 [file DataSheet_1.zip › Original images of blots修改/FIG7 C/TGFRII/GAPDH.tif]

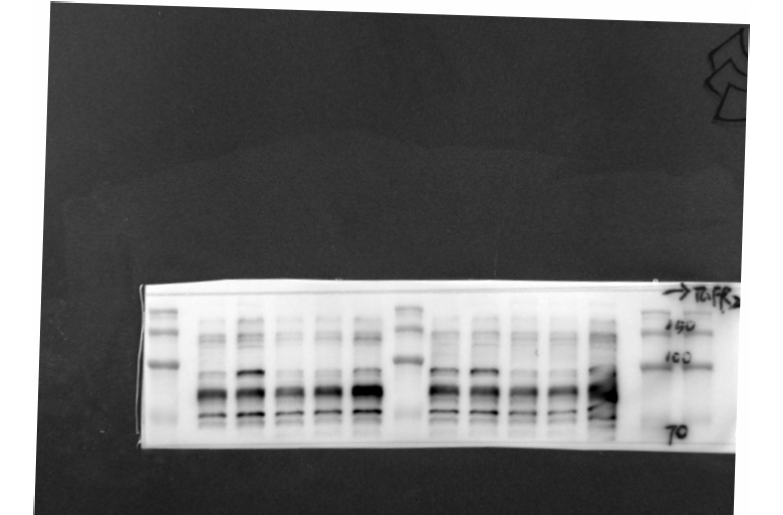

Supplement: Supplementary file 1 [file DataSheet_1.zip › Original images of blots修改/FIG7 C/TGFRII/TGFRII.tif]

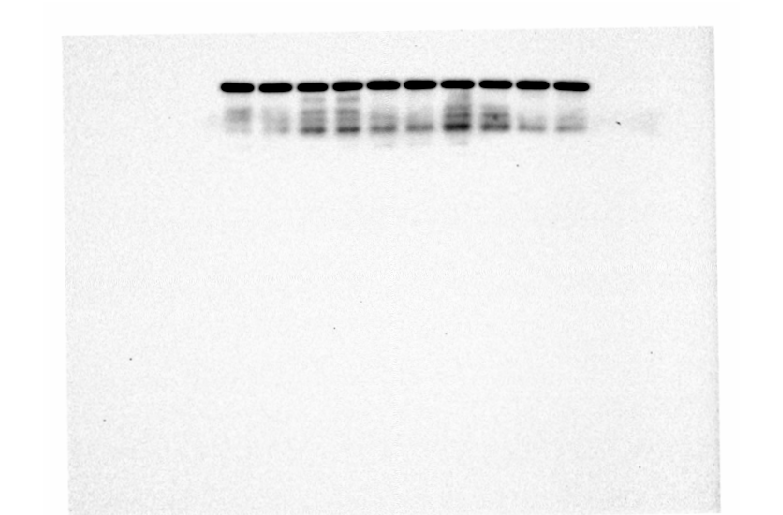

Supplement: Supplementary file 1 [file DataSheet_1.zip › Original images of blots修改/Supplementary Figure/GAPDH.tif]

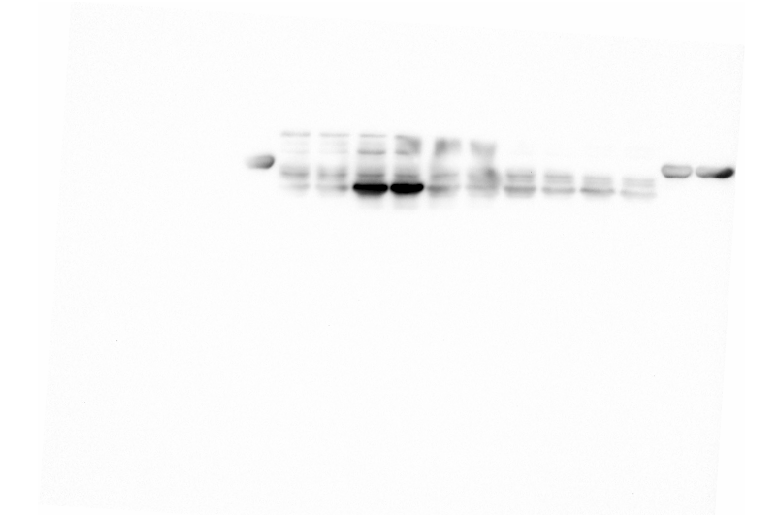

Supplement: Supplementary file 1 [file DataSheet_1.zip › Original images of blots修改/Supplementary Figure/mmp2.tif]

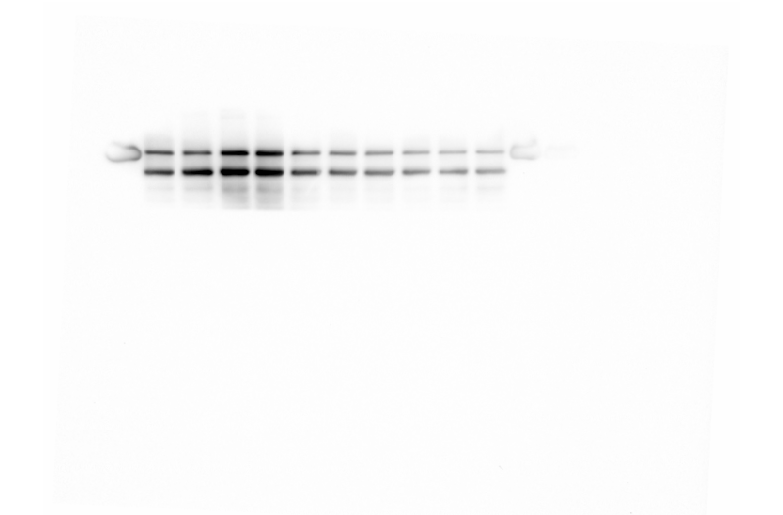

Supplement: Supplementary file 1 [file DataSheet_1.zip › Original images of blots修改/Supplementary Figure/MMP9.tif]

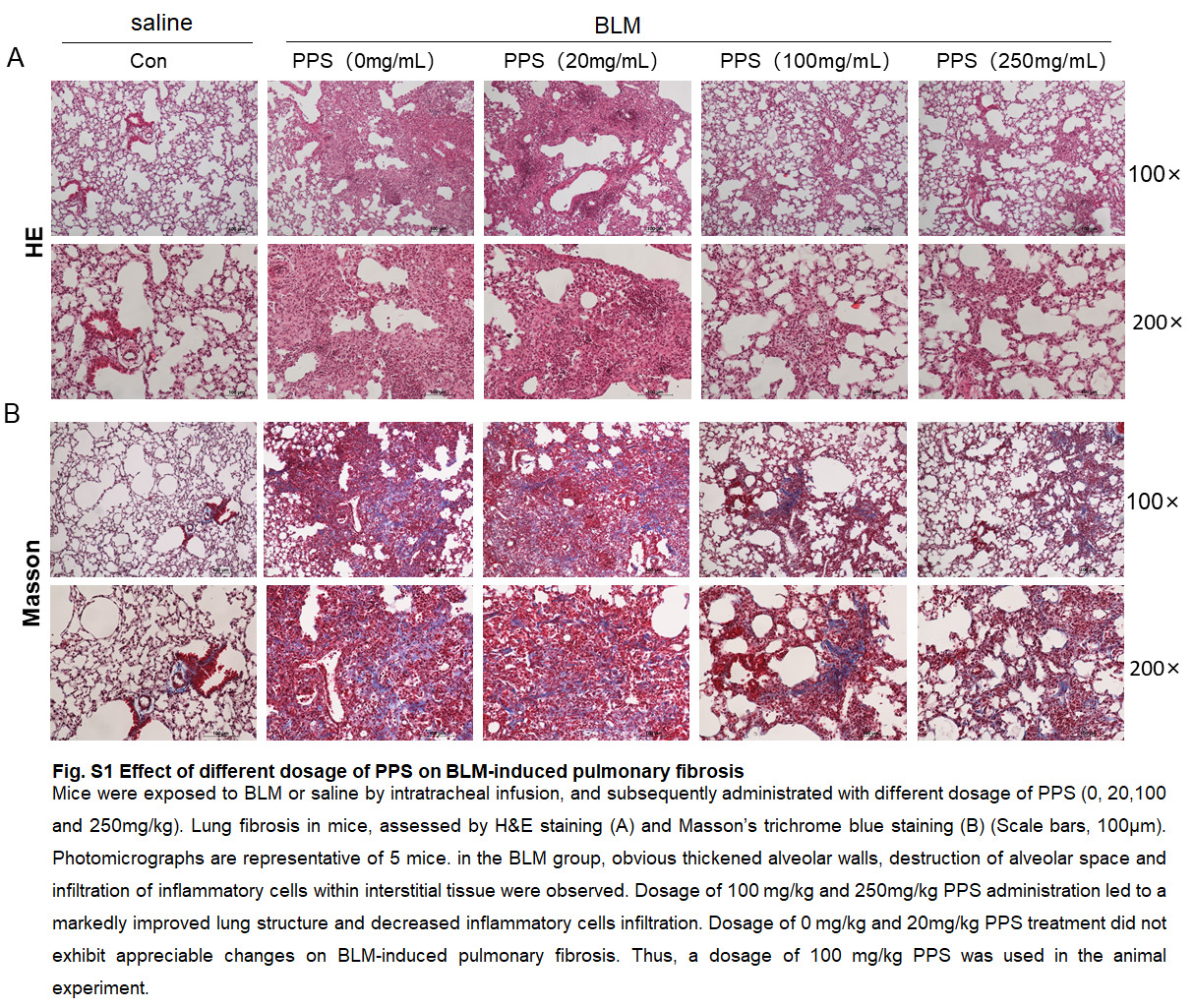

Supplement: Supplementary file 3 [file Image_1.tif]

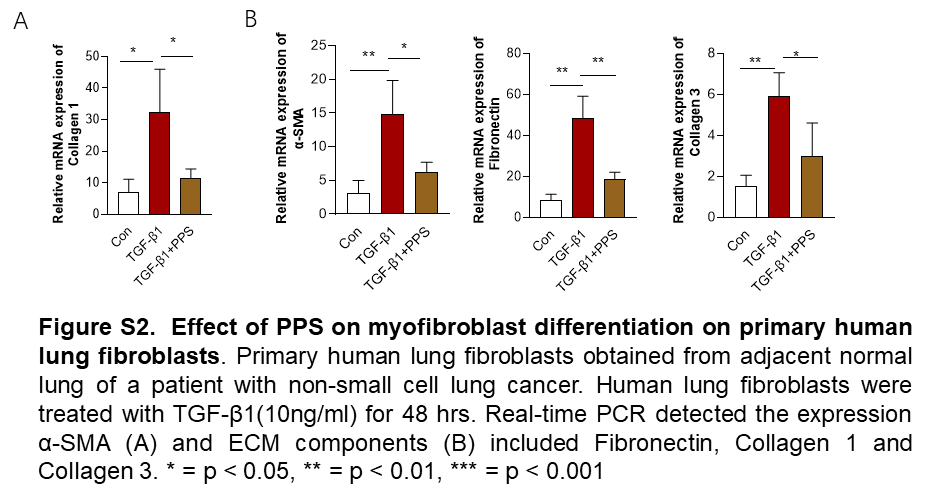

Supplement: Supplementary file 4 [file Image_2.tif]

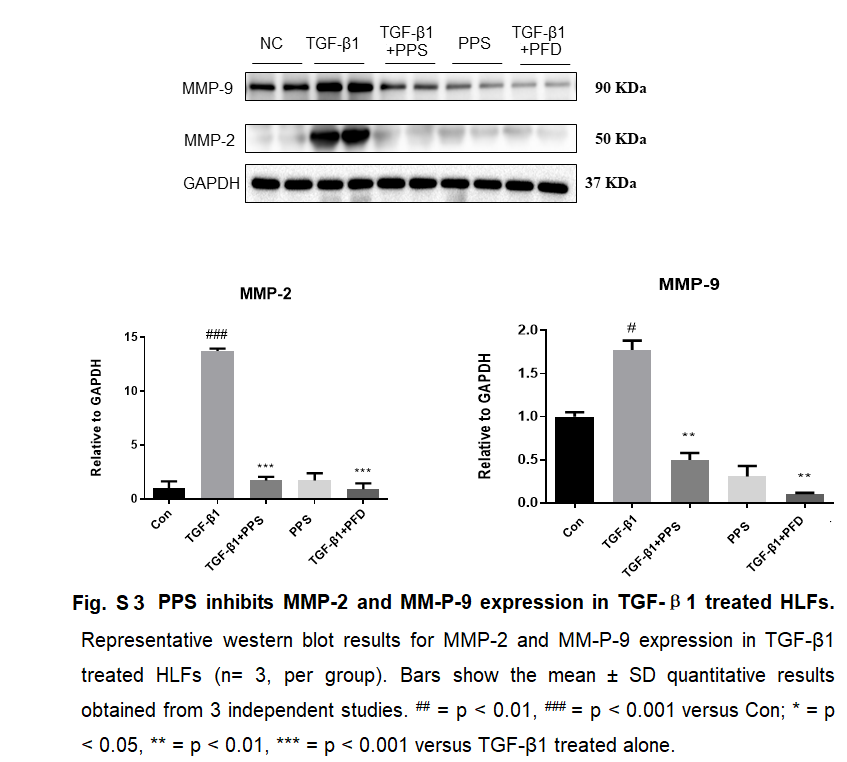

Supplement: Supplementary file 5 [file Image_3.tif]

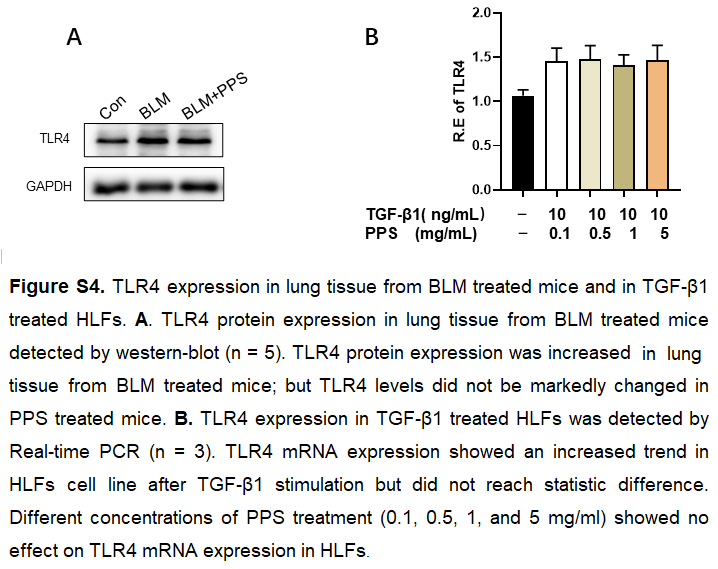

Supplement: Supplementary file 6 [file Image_4.tif]
