# Supplementary material for: Polyporus Polysaccharide Ameliorates Bleomycin-Induced Pulmonary Fibrosis by Suppressing Myofibroblast Differentiation via TGF-β/Smad2/3 Pathway
Source: Front Pharmacol. 2020 May 26;11:767. doi: 10.3389/fphar.2020.00767 (PMC7264095; doi:10.3389/fphar.2020.00767)
Supplement: Supplementary file 2 [file DataSheet_2.docx]

**Supplementary material**

**Table S1.** **Primary antibodies using in Western blot analysis**

| **Protein** | **Cat No.** | **Dilution** | **Company information** |
| --- | --- | --- | --- |
| fibronectin | 15613-1-AP | 1:1000 | Proteintech Group Inc., Chicago, IL, USA |
| collagen type 1 | 66761-1-1g | 1:1000 | Proteintech Group Inc., Chicago, IL, USA |
| collagen type 3 | 22734-1-AP | 1:1000 | Proteintech Group Inc., Chicago, IL, USA |
| Smad2 | 12570-1-AP | 1:1000 | Proteintech Group Inc., Chicago, IL, USA |
| GAPDH | 10494-1-AP | 1:2000 | Proteintech Group Inc., Chicago, IL, USA |
| α-SMA | 14395-1-AP | 1:1000 | Proteintech Group Inc., Chicago, IL, USA |
| Smad3 | 66516-1-1g | 1:1000 | Proteintech Group Inc., Chicago, IL, USA |
| TGF-β receptor Ⅱ | 66636-1-1g | 1:1000 | Proteintech Group Inc., Chicago, IL, USA |
| TGF-β [receptor](https://www.abcam.cn/tgf-beta-receptor-i-antibody-ab31013.html) Ⅰ | AF5347 | 1:1000 | Affinity biosciences,Inc., Cincinnati,OH, USA |
| phospho-Smad3 (Ser 213) | AF3366 | 1:1000 | Affinity biosciences,Inc., Cincinnati,OH, USA |
| phospho-Smad2 (Ser 255) | AF8059 | 1:1000 | Affinity biosciences,Inc., Cincinnati,OH, USA |
| MMP9 | 27306-1-AP | 1:1000 | Proteintech Group Inc., Chicago, IL, USA |
| MMP2 | 66366-1-Ig | 1:1000 | Proteintech Group Inc., Chicago, IL, USA |

**Table S2. Secondary antibodies using in Western blot analysis**

| **Product Name** | **Cat No.** | **Dilution** | **Company information** |
| --- | --- | --- | --- |
| HRP-Goat-anti-Rabbit IgG(H+L) | 111-035-003 | 1:5000 | Jackson ImmunoResearch Laboratories Inc.  Philadelphia, PA, USA |
| HRP-Goat-anti-mouse IgG(H+L) | 115-035-003 | 1:5000 | Jackson ImmunoResearch Laboratories Inc.  Philadelphia, PA, USA |

**Table S3. Human primers used for real-time quantitative PCR.**

| **Gene symbol** | **Forward (5′-3′)** | **Reverse (5′-3′)** |
| --- | --- | --- |
| fibronectin | TGCTCAACAGACAACCAA | CACCAGGACAGTAGAATCAG |
| collagen type I | AACTGGTACATCAGCAAGA | CTGGAATCCATCGGTCAT |
| collagen type Ⅲ | TTGAAGGAGGATGTTCCCATCT | ACAGACACATATTTGGCATGGTT |
| α-SMA | TTCCAGCCATCCTTCATC | ATTGTTAGCATAGAGGTCCTT |
| GAPDH | CGGAGTAACGGATTTGGTC | TGGGTGGAATCATATTGGAACAT |
